# Alpelisib: A Well-Tolerated Treatment Option for Patients with HR-Positive, HER2-Negative, PIK3CA-Mutated Advanced Breast Cancer

**DOI:** 10.1055/s-0041-1736239

**Published:** 2021-10-05

**Authors:** Fei-Yu Diao

**Affiliations:** 1 Department of General Surgery, Sun Yat-Sen Memorial Hospital, Sun Yat-Sen University, Guangzhou, People's Republic of China

In 2020, breast cancer replaced lung cancer as the most common cancer in the world. 1 As the world's most populous country, China contributes more than 11% of the global breast cancer cases in 2018. 2 3 The human epidermal growth factor receptor-2-negative (HER2 ^−^ ) luminal subtype (hormone receptor-positive [HR ^+^ ] and HER2 ^−^ ) represents approximately 70% of all breast cancer cases. 4 5 Endocrine-based therapy is the recommended initial treatment for HR ^+^ and HER2 ^−^ advanced breast cancer patients. 6 7 This treatment strategy can maintain the quality of life (QoL) for advanced breast cancer patients as long as possible before they switch to chemotherapy. Indeed, endocrine-based therapy combined with rapamycin inhibitors, CDK4/6 inhibitors, or PI3K inhibitors improves progression-free survival (PFS) of advanced breast cancer patients, and thereby delaying chemotherapy. 4 8 Therefore, adding the three types of targeted therapy drugs mentioned above to endocrine-based therapy before initiating chemotherapy is now considered the standard of care. Several previous studies have demonstrated that CDK4/6 inhibitors could maintain QoL in patients with advanced breast cancer. 9 However, QoL data in patients treated with PI3K inhibitors have not been reported. It is worth noting that approximately 40% of HR ^+^ and HER2 ^−^ breast cancer patients carry phosphatidylinositol-4,5-bisphosphate 3-kinase catalytic subunit α ( *PIK3CA* ) gene mutations. 4 10 In addition, these mutations are associated with a poor prognosis for advanced breast cancer patients. 10 11

SOLAR-1 is a randomized, phase III trial which compared alpelisib (a PI3K inhibitor) plus fulvestrant with placebo plus fulvestrant in patients with recurrence/progression of HR ^+^ and HER2 ^−^ advanced breast cancer who had received endocrine therapy (aromatase inhibitor-based treatment) previously. 4 12 It showed that alpelisib plus fulvestrant increased median PFS versus placebo plus fulvestrant (11.0 vs. 5.7 months). Unfortunately, as PI3Kα is also involved in normal human physiological processes, PI3Kα inhibitors can cause treatment-related adverse events, such as diarrhea, hyperglycemia, and rash. 4

Since the treatment for advanced breast cancer is palliative treatment, health-related QoL is the key factor in evaluating the risk-benefit status of treatment. 13 Patients with advanced breast cancer often experience pain and impaired QoL related to disease progression and treatment side effects. Pain and impaired QoL are important factors in treatment decisions for such patients. 14 Therefore, there is an urgent need to understand the effects of alpelisib on pain and QoL. In a study recently published in *Journal of Clinical Oncology* , titled “Patient-reported outcomes in patients with PIK3CA-mutated hormone receptor-positive, human epidermal growth factor receptor 2-negative advanced breast cancer from SOLAR-1,” Ciruelos and colleague 15 assessed the health-related QoL using standardized patient-reported outcomes measure in advanced breast cancer patients with *PIK3CA* mutations, HR ^+^ , and HER2 ^−^ who were enrolled in SOLAR-1 trial.

In this study, a total of 341 patients with *PIK3CA* mutations were enrolled and randomly assigned (1:1) to receive alpelisib plus fulvestrant or placebo plus fulvestrant. European Organization for Research and Treatment of Cancer QoL of Cancer Patients and Brief Pain Inventory-Short Form questionnaires were used to evaluate the patient-reported outcomes. Repeated measurement models and Cox models were used to analyze the alterations from baseline and time to 10% deterioration, respectively. Global Health Status/QoL and functional status were maintained from baseline in the alpelisib and placebo arms (overall change from baseline [95% confidence interval]: −3.50 [−8.02 to 1.02] for the alpelisib arm and 0.27 [−4.48 to 5.02] for the placebo arm). The overall treatment effect in Global Health Status/QoL was not statistically different between the alpelisib and placebo arms (−3.77; 95% confidence interval = −8.35 to 0.80; *p* = 0.101). Time to 10% deterioration for Global Health Status/QoL was not significantly different between arms (hazard ratio = 1.03; 95% confidence interval = 0.72–1.48). Compared with the patients treated with placebo, patients treated with alpelisib experienced deterioration in social functioning, diarrhea, loss of appetite, nausea or vomiting, and fatigue. Additionally, numerical improvement in worst pain was observed in the patients treated with alpelisib when compared with their placebo counterparts (42 vs. 32%; *p* = 0.090).

The SOLAR-1 study has proven that adding alpelisib to fulvestrant treatment can improve the median PFS of patients with PIK3CA-mutant advanced breast cancer. 4 Moreover, the present study demonstrated that fulvestrant plus alpelisib did not have a significant impact on QoL except with deterioration in social functioning as compared with fulvestrant alone. In addition, the worsening of pain and numeric improvement in worst pain in patients receiving the combination therapy of alpelisib and fulvestrant were delayed. However, patients who received alpelisib plus fulvestrant showed a slight decrease in social functioning subscale and symptom subscale scores (such as diarrhea and loss of appetite). The decline in these scores was basically consistent with the profiles of adverse event observed with the treatment of alpelisib plus fulvestrant. 4 This can be explained as diarrhea and loss of appetite may lead to a decline in social functioning. Since this study observed that the overall QoL of patients receiving alpelisib plus fulvestrant was not statistically different from that of patients received placebo plus fulvestrant, we speculated that the negative impact of adverse event-related symptoms on QoL was partly due to the delay in disease progression. Some patients enrolled in the SOLAR-1 trail discontinued the treatment of alpelisib or placebo because of adverse events, but continued fulvestrant treatment, 4 which might have also contributed to the delay in worsening of QoL. It is noteworthy that median PFS was shorter than time to 10% deterioration in each subscale. This finding indicates that the negative impact on functioning scores is mainly due to disease progression rather than study treatment.

This study has several limitations. Like all oncology studies that collect QoL questionnaires longitudinally, the main limitation of this study is missing data. In this regard, the authors followed United States National Research Council principles for analyzing incomplete data to assess the reasons for missing data and conduct a sensitivity analysis. They found that disease progression was the main reason for missing QoL data, followed by the discontinuation of study treatment due to treatment-related toxicity, but administrative reasons for missing data were considered to be non-informative. Another limitation relates to change over time in QoL. The 95% confidence interval for the change from baseline in the alpelisib arm ranged from −8.02 to 1.02, while that in the placebo arm ranged from −8.35 to 0.80. The difference between the two is −3.77. Although this study did not observe a statistical difference between alpelisib arm and placebo arm, the upper limit of the 95% confidence interval was extremely close to 0. However, the mean difference and the lower limit are both within 0 and 10 points, indicating that the magnitude of the difference was small. Both the significance and magnitude should be considered when evaluating treatment effects. Although this study still has the limitations mentioned above, this study still proved that the overall QoL of advanced breast cancer patients with HR ^+^ HER2 ^−^ and *PIK3CA* mutations was maintained with alpelisib plus fulvestrant treatment.
